# STAT3-mediated osteogenesis and osteoclastogenesis in osteoporosis

**DOI:** 10.1186/s12964-022-00924-1

**Published:** 2022-07-25

**Authors:** Xiaoli Hou, Faming Tian

**Affiliations:** School of Public Health, North China University of Science and Technology, Caofeidian Dis, Bohai Road 21, Tangshan, 063210 People’s Republic of China

**Keywords:** Osteoporosis, STAT3, Osteogenesis, Osteoclastogenesis

## Abstract

Osteoporosis is a common skeletal disease with marked bone loss, deterioration of the bone microstructure and bone fragility. An abnormal bone remodelling cycle with relatively increased bone resorption is the crucial pathophysiological mechanism. Bone remodelling is predominantly controlled by osteoblasts and osteoclasts, which are specialized cell types that are regulated by a variety of osteogenic and osteoclastic factors, including cytokines expressed within the bone microenvironment under local or systemic inflammatory conditions. Signal transducer and activator of transcription 3 (STAT3) plays a prominent role in the communication between cytokines and kinases by binding downstream gene promotors and is involved in a wide range of biological or pathological processes. Emerging evidence suggests that STAT3 and its network participate in bone remodelling and the development of osteoporosis, and this factor may be a potent target for osteoporosis treatment. This review focuses on the role and molecular mechanism of the STAT3 signalling pathway in osteogenesis, osteoclastogenesis and osteoporosis, particularly the bone-related cytokines that regulate the osteoblastic differentiation of bone marrow stromal cells and the osteoclastic differentiation of bone marrow macrophages by initiating STAT3 signalling. This review also examines the cellular interactions among immune cells, haematopoietic cells and osteoblastic/osteoclastic cells.

**Video abstract**

**Supplementary Information:**

The online version contains supplementary material available at 10.1186/s12964-022-00924-1.

## Background

As a serious public health problem, osteoporosis is a systemic bone metabolic disease characterized by reduced bone mass, low bone density and damaged bone microstructure and is accompanying an increased probability of fragility fractures [1, 2]. The incidence of osteoporosis is 70% in people aged over 80 years and 15% in people over 50 years [3]. At present, there are more than 200 million patients suffering from osteoporosis worldwide; as life expectancy increases globally, osteoporosis will lead to increased financial and medical burdens in most countries, and it is expected that the global cost of osteoporosis may increase to $131.5 billion by 2050 [4]. Fragility fractures caused by low-energy trauma, particularly hip fracture, are common complications of osteoporosis that result in poor health-related quality of life and a high risk of disability and mortality [5, 6]. Previous studies reported that more than 2 million hip fractures occurred worldwide in 2010, and 51% of these fractures could have been prevented if osteoporosis could be effectively treated [7]. The treatment for osteoporosis, thus far, mainly targets the balance between bone formation by osteoblasts and bone resorption by osteoclasts [8]. However, anti-resorption drugs such as bisphosphonates and the receptor activator of nuclear factor-κB ligand (RANKL) inhibitor denosumab or teriparatide, which is the only anabolic drug approved by the Food and Drug Administration (FDA), have or contribute to complicated side effects [9]. Thus, thoroughly clarifying the pathogenesis of osteoporosis, particularly the key molecules and underlying signalling pathways, and identifying new effective therapeutic targets for osteoporosis, is still challenging but important task.

The signal transducer and activator of transcription (STAT) family consists of seven proteins: STAT1, STAT2, STAT3, STAT4, STAT5α, STAT5β and STAT6 [10, 11]. These proteins are conserved among eukaryotes and are involved in a wide range of functions, such as cell differentiation, cell proliferation, the immune response, cell survival, apoptosis, and angiogenesis [12, 13]. Among this family of latent cytoplasmic protein members, STAT3 plays a prominent role in the communication between cytokines and kinases. Normal STAT3 signalling is tightly controlled during the standard cellular response. Suppressors of cytokine signalling (SOCS) proteins and protein tyrosine phosphatases (PTPs) provide negative feedback to the receptor and prevent continuous signalling by switching off the signalling cascade under physiological conditions [14]. Currently, the phosphorylation of STAT3 has been proven to be a crucial event in several specific signalling networks that regulate osteogenic and osteoclastic processes.

This review focuses on the role of STAT3 in regulating the balance of bone remodelling in osteoporosis, particularly the differentiation and function of osteoblasts and osteoclasts. We also discuss recent advances in how STAT3 links other molecules to regulate osteoblastic differentiation in bone marrow stromal cells (BMSCs) and osteoclastic differentiation in bone marrow macrophages (BMMs), thereby affecting bone remodelling, as well as the interactions among immune cells, haematopoietic cells and bone cells.

## Pathological mechanism of osteoporosis

Postmenopausal osteoporosis and senile osteoporosis are two main pathophysiological processes that are related to significant bone loss [15, 16]. While there are various causes of osteoporosis [17], the uncoupling of the bone remodelling cycle and increased bone resorption relative to formation are common underlying pathophysiological mechanisms. Bone remodelling starts with the destruction of old bone by osteoclasts followed by the deposition of osteoids by osteoblasts within basic multicellular units (BMUs), which are composed of osteoclasts, osteoblasts and the capillary blood supply. Subsequently, the organic extracellular matrix is mineralized [16, 18]. This process, which occurs throughout life, may replace microdamage and ensure structural integrity. However, imbalanced bone remodelling with increased resorption relative to formation could lead to osteopenia or even osteoporosis [16, 19]. In this context, several factors have been shown to impact bone remodelling and reduce bone mass: (1) changes in the differentiation of mesenchymal stem cells with more adipocytes and fewer osteoblasts [20]; (2) vitamin D deficiency leading to secondary hyperparathyroidism, which in turn enhances osteoclastic bone resorption [21, 22]; (3) altered immune cell activity and elevated inflammatory cytokines such as IL-1, IL-6 and IL-11 [23]; and (4) a decline in oestrogen levels, which stimulates the expression of RANKL, which activates osteoclasts, especially in postmenopausal women [24]. Interestingly, under oestrogen-deficient conditions, the crosstalk between oestrogen and bone cell-produced cytokines tends to cause bone loss; for example, IL-6 stimulates bone resorption, whereas oestrogen blocks osteoblast synthesis of IL-6 and antagonizes the IL-6 receptor [25]. These factors contribute to bone loss and deterioration of the bone microarchitecture, while the underlying mechanism, particularly the cellular and molecular details of the interactions among these factors, remains unclear.

## Structure and signalling cascades of STAT3

**Fig. 1:**
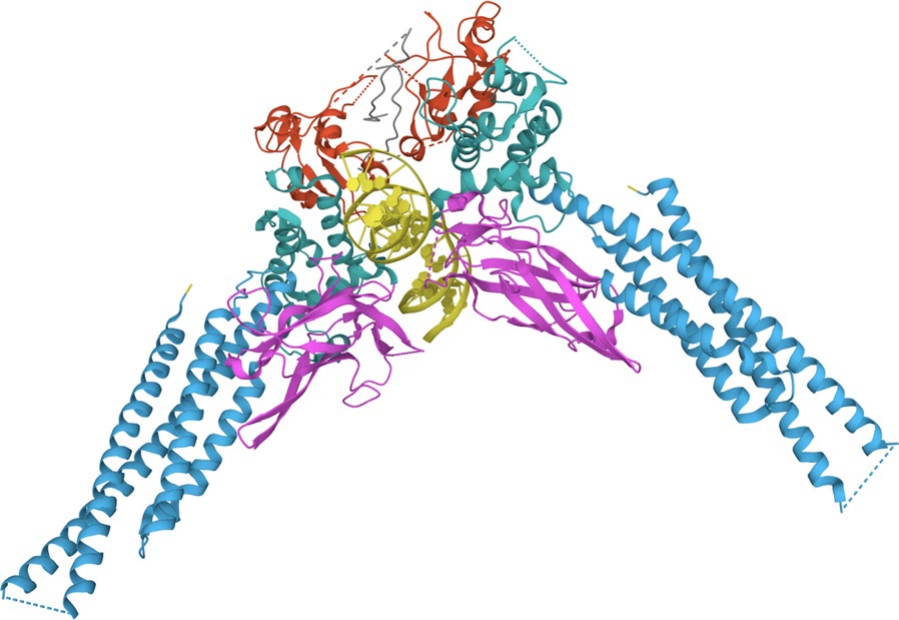
Crystal structure of a STAT3128–715, monomer as follows: blue CCD (138–320), magenta DBD (321–465), green LD (466–554), red SH2 (586–688), and partially TAD in grey (689–715). Missing residues are displayed as dash lines. The PDB entry is 6QHD

STAT3, which has a relative molecular weight of 92 kDa, consists of approximately 770 amino acids and includes six functional domains: the N-terminal domain, coiled-coil domain, DNA binding domain, C-terminal transactivation domain, linker domain, and Src homology 2 (SH2) domain. Each domain which plays a different role in signal transduction and the activation of gene transcription [26, 27]. The N-terminal domain is involved in STAT3 dimer nuclear translocation, synergistic DNA binding, protein–protein interactions to form various dimer complexes, and subsequent negative regulatory processes [27]. The coiled-coil domain is responsible for mediating the interaction of STAT3 with other proteins. The DNA binding domain recognizes specific DNA sequences and provides a binding interface between proteins and DNA to form STAT3-DNA complexes [13]. The linker domain is involved in interactions with the transcriptional system [11]. Because STAT3 is a regulatory module for intracellular signalling cascades, the SH2 domain is critical for STAT3 binding to kinase receptor residues and dimerization. The SH2 domain acts in a sequence-specific and strictly phosphorylation-dependent manner through highly affinity interactions with phosphorylated tyrosine-containing target peptides that are on the cytoplasmic side of cytokine receptors, resulting in subsequent tyrosine kinase phosphorylation of STAT3 [11]. Phosphorylated STAT3 can form homodimers and STAT1-STAT3 heterodimers through interactions between the phospho-tyrosine motif of one and the SH2 domain of the other [28]. The STAT3 SH2 domain contains three important solvent-accessible subpockets, which can be targeted by small-molecule inhibitors and are potential therapeutic targets [29, 30]. In addition, STAT3 has two phosphorylation sites: tyrosine 705 (Tyr705) and serine 727 (Ser727). The transcriptional role of STAT3 is canonically regulated by tyrosine phosphorylation [31]. Tyr705 phosphorylation is essential for p-STAT dimerization, which in turn mediates nuclear translocation, DNA binding, and target gene expression [12, 32]. The phosphorylation of Tyr705 is mainly mediated by receptor-associated JAK kinases, Src family kinases, or receptor type tyrosine kinases, and tyrosine-phosphorylated STAT3 forms a stable dimer by reciprocal interactions between the phospho-Tyr705 (pY705)-containing region and the SH2 domain of the other STAT3 molecule [33]. Ser727 phosphorylation has been considered to be a secondary event after Tyr705 phosphorylation. The C-terminal Ser727 is phosphorylated by multiple Ser/Thr kinases, and phospho-Ser727 (pS727) has been shown to enhance STAT3 transcriptional activity. Interestingly, in mitochondria, STAT3 requires pS727 but not pY705 to enhance electron respiratory chain activity and Ras-dependent tumorigenesis [34, 35] (Fig. 1).

The canonical STAT3 signalling pathway involves JAKs/STAT3, including the glycoprotein (gp130) receptor (also called IL-6 Rβ), Janus kinases (JAKs) and STAT3. The signalling is triggered by the binding of IL-6 to the IL-6 receptor (IL-6R), which forms the IL-6/IL6R complex that activates a homodimer of gp130, which is the signal transducer for all cytokines in the IL-6 family [36, 37]. Following receptor dimerization, receptor-coupled JAKs are close to each other and activated by tyrosine phosphorylation. The activated JAKs phosphorylate multiple tyrosine residues on gp130 receptors and activate the STAT1/STAT3, phosphatidylinositol-4,5-bisphosphate 3-kinase (PI3K)/AKT and Src homology region 2 (SH-2) domain-containing phosphatase 1 and 2 (SHP2)/renin-angiotensin system (RAS)/extracellular signal-regulated kinase (ERK) signalling cascade, as well as STAT3-mediated induction of the negative regulator SOCS3 [38]. The phosphorylated tyrosine residue, which is a docking site, recruits cytoplasmic STAT3 and binds inactive STAT3 through the SH2 domain. After binding to gp130, STAT3 is phosphorylated by JAK at Tyr705, resulting in STAT3 dimerization and an increase in nuclear concentrations. Subsequently, the activated dimer exposes a nuclear localization signal and translocates into the nucleus via import systems such as importin-a/importin-b1-Ran, after which it binds to the promoters of target genes to modulates their transcription. These target genes are involved in cell differentiation, proliferation and survival, as well as apoptosis, angiogenesis and inflammation [31, 39–42].

STAT3 function is tightly regulated by endogenous inhibitors such as SOCS3 and PTP proteins and inhibitors of activated STATs (PIASs). SOCS3, which belongs to the SOCS family, consists of an SH2 domain and a segment called the SOCS box near the C-terminus and can regulate signal transduction by inhibiting the components of the JAK/STAT3 pathway. The SH2 domain of SOCS3 binds to SHP-2-binding domains of the gp130 receptor, accessing the activation loop of JAK through its kinase inhibitor region domain to inhibit JAK activity [43]. Dephosphorylation of the tyrosine residue by PTPs is the main negative regulatory mechanism of STAT3. Currently, seven PTPs have been shown to dephosphorylate STAT3 and regulate STAT3 signalling: PTP receptor-type D, T and K (PTPRD, PTPRT, PTPRK), SHP1, SHP2, MEG2/PTP nonreceptor type 9 (PTPN9) and T-cell PTP (TC-PTP)/PTP nonreceptor type 2 (PTPN2) [44]. PTPRD, PTPRT, PTPRK and PTPN9 negatively regulate STAT3-mediated signalling by directly dephosphorylating STAT3 at Tyr705. SHP1 reduces STAT3 phosphorylation in coordination with SHP2 and TC-PTP [45, 46]. Furthermore, in response to cytokine stimulation, protein inhibitor of activated STAT3 (PIAS3) binds to activated STAT3 and prevents it from binding to DNA [47].

Thus, the structure-based function and modification of STAT3 is crucial, and its involvement in a series of biological and pathological processes has drawn increasing attention. Interestingly, STAT3 can play different or even opposite roles in various conditions, depending on cell type, developmental status and stimulus. The most prominent and well-studied role of STAT3 in cancer is as a proto-oncogene. Aberrant STAT3 activity has been detected in a variety of solid and haematological tumours [48, 49]. However, Grabner et al. demonstrated that STAT3 played a -suppressive role in V-Ki-ras2 Kirsten rat sarcoma viral oncogene homologue (KRAS) mutant lung adenocarcinoma [50]. The dual roles of STAT3 have also been identified in glioblastoma, in which STAT3 exerts both tumour-enhancing and tumour-suppressive effects depending on the genomic mutational profile [51]. Notably, the expression of the STATS family has been identified in bone tissues, especially STAT3, which has been reported to be involved in cell proliferation, apoptosis and the motility of osteoblasts and osteoclasts, the crosstalk between which plays a crucial role in bone remodelling and related bone diseases.

## STAT3 signalling pathway and osteogenesis

BMSCs may differentiate into osteogenic, adipogenic, myogenic, and chondrogenic lineages. The commitment of BMSCs to becoming osteoblasts is crucial for osteogenesis; when immature osteoblasts mature and become functional, they begin to embed in the bone as osteocytes and regulate matrix mineralization and bone quality [52]. The balance between osteoblast proliferation and apoptosis determines the number of active osteoblasts and thus affects the homeostasis of bone formation [53].

### Crucial role of osteoblast lineage cell produced STAT3 in osteogenesis

Regarding the role of STAT3 in bone, a number of in vivo studies using cell-specific STAT3 transgenic mice were performed. Itoh et al. found a reduction in the bone formation rate in the osteoblast-specific STAT3-knockdown mice (α1(I)^Cre^/STAT3^flox/−^, heterozygous floxed STAT3 and expressing Cre under the α1(I) promoter), which indicates that STAT3 signalling is important for osteoblast function. Recently, Zhou et al. proved that ablation of STAT3 in BMSCs resulted in skeletal deformities, as osteoblast lineage-specific STAT3-knockout mice (Prx1^Cre^; STAT3^fl/fl^, homozygosis floxed STAT3 and expressing Cre under Prx1 promoter) were dwarfed relative to their control littermates, whereas in preosteoblast-specific STAT3-knockout mice (Osx^Cre^; STAT3^fl/fl^, homozygosis floxed STAT3 and expressing Cre under the Osterix (Osx) promoter) exhibited an osteoporotic phenotype with reductions in bone volume/tissue volume (BV/TV), Tb.Th, Tb.N and cortical thickness (Ct.Th) but an increase in Tb.Sp [54] (Tables 1, 2).

**Table 1:** Phenotypes of transgenic mice with cell-specific modified STAT3

Cell	Transgenic strategy	Effect on STAT3	Phenotypes	Reference
Osteoblast linage cells	α1(I)Cre/STAT3^flox/−^ (10-week-old)	Disruption of STAT3 in osteoblast	Increased mineral apposition rate	[55]
	Prx1 ^Cre^; STAT3^fl/+^ mice (8-week-old)	Deletion of STAT3 in BMSCs	Dwarfed; defects and malformation in the skeleton of new-borns	[54]
	Osx^Cre^; STAT3^fl/+^ (8-week-old)	Deletion of STAT3 in pre-osteoblast	Dwarfed; hypo mineralization	[54]
Osteoclast linage cells	STAT3^Ctsk^ (20-week-old)	Knockout STAT3 in osteoclast cells	Increased bone mass	[83]
	STAT3^Ctsk^ (8-week-old)	Knockout STAT3 in osteoclast cells	Lower BMD in female femurs	[84]
Hematopoietic cells	Hematopoietic cell-specific disruption of the STAT3	Tissue-specific STAT3 deletion in hematopoietic bone marrow cell lineages	Decreased bone mass and enhanced osteoclast function	[101]

**Table 2:** Phenotypes of osteoblast linage cells with modified STAT3 signaling pathway

Origin	Signaling pathway	Regulating factors	Effect on STAT3	Downstream target gene	Phenotypes	Reference
Mice BMSCs	JAK/STAT3	LIF	Increased STAT3 phosphorylation	OCN, BSP, ALP, COLIαI, RUNX2, OSX	Suppressed osteogenic differentiation	[43]
SHP2KO^Bglap^ osteoblast cells	RUNX2/OSTERIX, SHP2/STAT3	SHP2	Increased STAT3 phosphorylation	RANKL	Inhibited cell maturation	[52]
gp130^FXXQ/FXXQ^ mice osteoblast	YXXQ/STAT3-dependent	IL-6	Decreased STAT3 phosphorylation	ALP	Decreased mineralization	[55]
gp130^F759/F759^ mice osteoblast	Y759/SHP-2-dependent negative regulatory	IL-6	Increased STAT3 phosphorylation	ALP	Increased cell differentiation	[55]
Primary bone-derived cells	JAK2/STAT3	IL-17A	Increased STAT3 phosphorylation	ALP	Increased cell differentiation and mineralized nodes	[56]
Human primary osteoblastic cells	JAK2/STAT3	PC1-CT	Increased STAT3 phosphorylation; STAT3-DNA binding	RUNX2	Increased cell differentiation	[60]
hFOB 1.19	JAK2/STAT3	EPO	Increased STAT3 phosphorylation	ALP, OCN, OPG, OPN	Stimulated osteoblast proliferation and differentiation	[70]
MC3T3-E1 cells	JAK3/STAT3	NDRG2	Increased STAT3 phosphorylation	RUNX2, OPG, OSX, ALP, OCN	Increased cell differentiation and	[62]
Osx::PKD1^fl/fl^ calvarial preosteoblasts	STAT3	PKD1	Increased STAT3 phosphorylation	ALP, OSX, RUNX2, COL-α1	Increased cell differentiation	[64]
Mice MSCs	STAT3	HIF-1α	Increased STAT3 phosphorylation	COL1α1, RUNX2, ALP, OSX, OCN, VEGF	Increased cell differentiation and mineralization	[78]
MC3T3-E1 cells	SOCS3/STAT3	CUEDC2	Decreased STAT3 phosphorylation	ALP, RUNX2, OSX	Decreased cell differentiation	[65]
Prx1^Cre^; Lepr^fl/fl^ mice SSCs	JAK2/STAT3	LepR	Decreased STAT3 phosphorylation		Blocked adipocyte differentiation;	[61]
Human BMSCs	JAK1/STAT3	RPN2	Decreased STAT3 phosphorylation and nuclear location	OCN, OPN, RUNX2, BSP, ALP	Increased osteogenic differentiation and mineralized nodes	[67]
ΔTsc1 primary calvarial cells	STAT3/p63/Jagged/Notch	mTOR1	Increased STAT3 phosphorylation	RUNX2	Prevented osteoblast maturation and mineralization	[68]
MC3T3-E1 cells	JAK2/STAT3	miR-135b	Decreased JAK2 STAT3 phosphorylation	ALP	Decreased cell viability, and mineralized nodes; increased cell apoptosis	[72]
Human MSCs	STAT3/miR-7-5p/CRY2 CLOCK, BMAL1/ P300		Increased STAT3 phosphorylation	ALP, RUNX2, OCN, TYPE I COLLAGEN	Increased cell differentiation and mineralization	[74]
MC3T3-E1 cells	STAT3	miR-3074-5p,	Decreased STAT3 phosphorylation	XIAP, c-IAP2, SURVIVIN,	Promoted cell apoptosis	[73]
Human MSCs	JAK/STAT3	miR‐224	Decreased STAT3 phosphorylation	OCN, OPN, RUNX2, BSP, and ALP	Increased cell differentiation and mineralization	[77]
Human BMSCs	JAK/STAT3	OSM	Increased STAT3 phosphorylation	ALP, RUNX2	Increased cell differentiation and mineralized nodes	[109]
Vascular smooth muscle cells	STAT3/ Runx2	IL-6/IL-6R	Increased STAT3 phosphorylation	RUNX2, ALP, OPN	Increased cell osteoblast-like differentiation and mineralization	[110]

These cell-specific knockout or knockdown strategies showed that that STAT3 was necessary and essential for bone formation and development and had a somewhat more vital impact when the deficiency occurred in a more nascent linage. Thus, whether the deletion of STAT3 in mature osteoblasts using osteoblast-specific STAT3-knockout mice (Ocn^Cre^; STAT3^fl/fl^, homozygosis floxed STAT3 and expressing Cre under the Osteocalcin (Ocn) promoter) would also affect bone formation or cause skeletal deformities is still unknown.

### Proteins regulating osteogenesis via STAT3 activation

To uncover the role of STAT3 in osteogenesis with or without specific stimulation, increasing numbers of in vivo and in vitro studies have been performed. Although most studies have shown that many proteins contribute to osteogenic differentiation and promote osteogenesis by activating STAT3, some proteins have been shown to stimulate osteogenesis by inhibiting the activation of STAT3. Otherwise, p-STAT3 could regulate downstream genes and signalling pathways, subsequently suppressing osteoblastic differentiation and osteogenesis. We reviewed the cell-specific effects and mechanisms with respect to the role of STAT3 in osteogenesis.

#### Cytokines

**Fig. 2:**
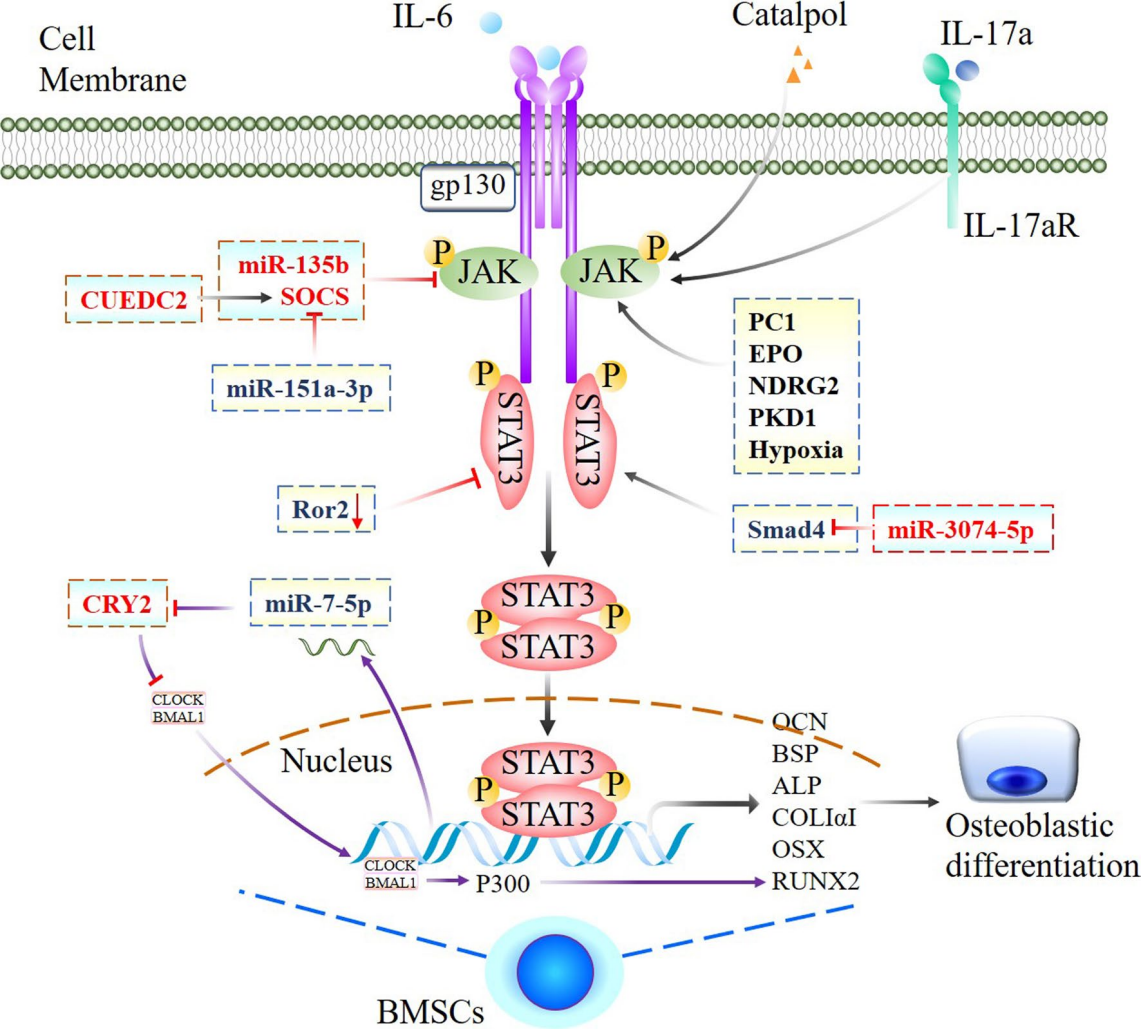
Jak2/STAT3 signal network positively regulates osteogenic differentiation. Proposed model of the role of JAK/STAT3 mediated network that positively regulating osteogenic differentiation of osteoblast precursors. A series of cytokines, molecules including some miRNAs interacted with JAK/STAT3 axis to regulate downstream effectors take account for osteogenic differentiation, all marked with event-specific colored line or arrows. Those delineated in a rectangle with dotted line indicate functional similarity. Ending with red bars indicate inhibition or downregulation, arrows indicate positive stimulation or upregulation

IL-6 family cytokines act on osteoblasts and induce the differentiation of osteoblasts or osteoclasts by binding to the gp130 receptor, which is involved in multiple signalling pathways, such as YXXQ-dependent STAT3 signalling and Y759-dependent signalling [55]. Compared with the gp130^WT/WT^ mice, mutant gp130 knock-in (mice gp130^F759/F759^), which lack the Y759/SHP-2-dependent negative regulatory signal, exhibited an osteosclerotic phenotype with higher bone volume (BV), trabecular number (Tb.N), and trabecular thickness (Tb.Th) and lower trabecular separation (Tb.Sp). Conversely, the activities of alkaline phosphatase (ALP) and mineralization induced by IL-6 family cytokines were significantly decreased in mutant gp130 knock-in mice (gp130^FXXQ/FXXQ^), in which the YXXQ/STAT3-dependent signal was selectively disrupted. These results revealed that STAT3 signal activation by gp130 positively regulates osteoblastic differentiation to promote bone formation [55]. IL-17A, which is the most abundant cytokine in ankylosing spondylitis sera and synovial fluid, enhanced osteoblast activity by increasing phosphorylated JAK2 (p-JAK2), total JAK2, and phospho-STAT3 (Tyr705) signalling. The addition of exogenous IL-17A to primary bone-derived cells (BdCs) promoted the osteogenic stimulus-induced increase in ALP activity and mineralization. Blocking IL-17A attenuated ALP activity and mineralization in BdCs by inhibiting JAK2 phosphorylation and downregulating the expression osteoblast-associated genes [56] (Tables 1, 2; Fig. 2).

**Fig. 3:**
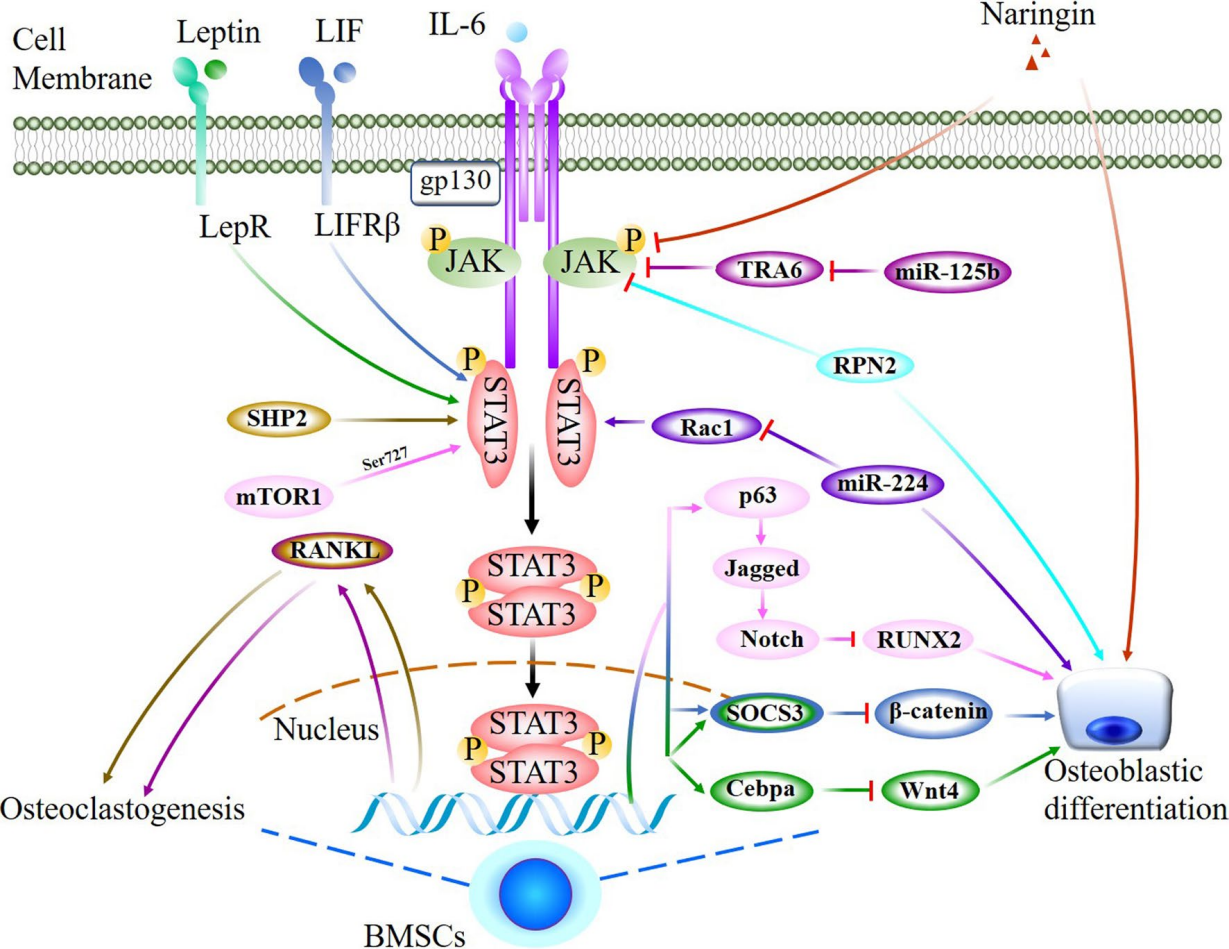
Jak2/STAT3 signal network negatively regulates osteogenic differentiation. JAK/STAT3 mediated signaling pathway and interlinked network that negatively regulation of osteogenesis via regulating activations of JAK and/or STAT3, a complicated and stimulator-specific role of STAT3 were indicated and marked with event-specific colored line or arrows. Ending with red bars indicate inhibition or downregulation, arrows indicate positive stimulation or upregulation

Leukaemia inhibitory factor (LIF) is a pleiotropic cytokine that belongs to the IL-6 family and has a suppressive effect on osteoblast differentiation [57]. By binding to the receptors gp130 and LIFRꞵ, LIF activates the JAK3/STAT3 signalling pathway, promotes the expression of SOCS3 and induces the interaction of SOCS3 and ꞵ-catenin, reducing ꞵ-catenin protein levels and suppressing osteoblastic differentiation [43]. Similarly, an in vitro study demonstrated that LIF was implicated in the high glucose (HG)-mediated inhibition of osteoblast differentiation in human osteosarcoma MG-63 cells by promoting STAT3/SOCS3 signalling, as the downregulation of osteogenic differentiation markers by LIF was restored by a STAT3 inhibitor [58] (Table 2; Fig. 3).

#### Transmembrane proteins

The ability of BMSCs to differentiate into osteoblasts requires Runt-related transcription factor 2 (Runx2) expression, which has been proven to be regulated by STAT3. P-STAT3 translocates to the nucleus, where it binds to the Runx2 promoter and enhances transcription, thereby triggering osteoblastic differentiation in BMSCs [59, 60]. In human osteoblastic cells, Polycystin-1 (PC1) is a transmembrane mechanosensor that senses extracellular mechanical cues and generating a response through cleavage of its C-terminal cytoplasmic tail (CT). The PC1-CT in turn triggers JAK2/STAT3 activation and promotes Runx2 transcription, inducing bone formation [60] (Table 2; Fig. 2).

However, as a nutritional/adiposity sensor, Leptin receptor (LepR) acts on mesenchymal stromal cells in the bone marrow to increase adipogenesis and reduce osteogenesis by enhancing the activation of JAK2/STAT3 signalling. Two weeks after bone fracture, micro-CT analysis showed significantly increased trabecular BV, Tb.N, and Tb.Th and reduced Tb.Sp in osteoblast lineage-specific Lepr-knockout mice (Prx1-Cre; Lepr^fl/fl^; homozygosis floxed STAT3 and expressing Cre under the Prx1 promoter). Femurs from osteoblast lineage-specific JAK2-activated mice (Prx1-Cre; JAK2^V617F^; JAK2 activated and expressing Cre under the Prx1 promoter) exhibited a significant reduction in femur length and trabecular BV. In vitro, a dramatic reduction in phospho-STAT3 (Tyr705) levels was observed in PDGFRα^+^CD45^−^Ter119^−^CD31^−^ skeletal stem cells (SSCs) from Prx1-Cre; Lepr^fl/fl^ mice compared to control mice [61] (Tables 1, 2; Fig. 3).

#### Cytoplasmic proteins

N-myc downstream regulator gene 2 (NDRG2) is involved in cell proliferation, differentiation and hormone response. Chen et al. studied the role of NDRG2 and found that NDRG2 overexpression promoted bone morphogenetic protein 2 (BMP2)-induced osteoblastic differentiation by increasing the expression of Runx2, OPG, OSX and OCN through the JAK3/STAT3 signalling pathway [62]. The protein kinase D (PKD) family of serine/threonine kinases has been implicated in the regulation of bone biology. Heterozygous animals with attenuated PKD1 activity (PRKD1^±^ mice) exhibit decreased bone mass and impaired osteoblast function during pubertal growth [63]. Furthermore, compared with littermate controls, preosteoblast-specific PKD1-knockout mice (Osx^Cre^; PKD1^fl/fl^; homozygosis floxed PKD1 and expressing Cre under the Osx promoter) showed significantly lower bone mass and deterioration of the microarchitecture. Mechanistically, silencing endogenous PKD1 reduced osteoblast markers, such as Runx2, OSX, OPN and OCN, in a preosteoblast cell line (MC3T3-E1 cells), impaired osteoblast differentiation and decreased the phosphorylation of STAT3 (Ser727) and p38, whereas the overexpression of PKD1 upregulated the expression of osteoblastic markers and STAT3 and p38 phosphorylation [64]. JAK/STAT3 signalling is negatively regulated by SOCS3, as mentioned previously. CUE domain-containing 2 (CUEDC2), which is involved in ubiquitin-mediated protein degradation, binds to SOCS3 and reduces the ubiquitination of SOCS3. CUEDC2 has been shown to inhibit osteoblast differentiation and bone formation by inhibiting STAT3 activation through interactions with SOCS3. Knockdown of CUEDC2 increased the phosphorylation of STAT3 in the cytosol of MC3T3-E1 cells. Conversely, treatment with Stattic, an inhibitor of STAT3, significantly inhibited Ad-shCUEDC2-induced osteoblast differentiation in a dose-dependent manner [65]. The receptor tyrosine kinase-like orphan receptor 2 (Ror2) promoter region contains a GAS motif, which is thought to be a cis element for STATs. An experiment confirmed that Ror2 deficiency impaired osteogenesis in mouse BMSCs by deactivating STAT3, but whether Ror2 and STAT3 interact directly during mouse BMSC osteogenesis remains unclear [66] (Tables 1, 2; Fig. 2).

However, the highly conserved glycoprotein Ribophorin II (RPN2) is present in the rough endoplasmic reticulum membrane and participates in multiple biological reactions. RPN2 overexpression promoted hBMSCs osteogenic differentiation and markedly reduced the expression of JAK1 and p-STAT3 by inducing JAK1 ubiquitination. Silencing JAK1 could rescue the decreased expression of osteogenic genes caused by reducing RPN2, indicating an inhibitory effect of JAK1/STAT3 on osteogenic differentiation [67]. Mechanistic target of rapamycin complex 1 (mTOR1) regulates cell growth and metabolism by integrating both intracellular and extracellular signals. Preosteoblast-specific Tsc1-knockout mice (Osx-GFP::Cre^TG/+^; Tsc1^flox/flox^ (ΔTsc1); homozygosis floxed Tsc1 and expressing the GFP-Cre fusion protein under the Osx1 promoter) showed marked increases in bone mass in distal regions of the femur, and the BV/TV, Tb.N, Tb.Th increased by 240%, 140% and 170%, respectively, while the Tb.Sp decreased by 50%. In vitro, mTOR1 induced the phosphorylation of STAT3 at Ser727, and phosphorylated STAT3 bound to the p63 promoter in osteoblasts. P63 is a positive regulator of Jagged expression and Notch activity. mTORC1 inhibits osteoblast maturation by activating the STAT3/p63/Jagged/Notch pathway and downregulating Runx2 [68]. SHP2 is a ubiquitously expressed protein tyrosine phosphatase (PTPase) that plays an essential role in osteogenesis and bone mineral homeostasis. BV/TV, connectivity density, and Tb.N. were markedly reduced in the proximal tibiae of 10-week-old osteoblast-specific SHP2 knockout-mice (SHP2KO^Bglap^ mice; homozygosis floxed SHP2 and expressing Cre under the Bglap promoter) compared with age- and sex-matched SHP2CTR^Bglap^ littermate controls, while the structure model index, Tb.Sp. and bone marrow cavity were significantly increased. SHP2 deficiency promoted RANKL production in osteoblasts and osteocytes by promoting STAT3 Tyr705 phosphorylation, followed by local osteoclastogenesis and mineral resorption [52] (Tables 1, 2; Fig. 3).

### Drugs that regulate osteogenesis via STAT3 activation

Following the development of osteoporosis treatments, the use of traditional Chinese medicine to treat osteoporosis has attracted extensive attention in recent years. A number of traditional Chinese medicines inhibit osteoporosis through the JAK2/STAT3 pathway. Catalpol is a natural iridoid glycoside that is mainly enriched in the dried root of Rehmanniae and possesses a variety of biological activities, such as anti-ischaemic, antioxidative and anti-inflammatory effects. Catalpol facilitated bone regeneration and vessel formation in a calvarial defect rat model with OVX-induced osteoporosis and promoted the osteogenic ability of BMSCs and BMSC-dependent angiogenesis through by activating the JAK2/STAT3 axis; these benefits could be partially abolished by knocking down STAT3 [69]. Erythropoietin (EPO) is a glycoprotein that can promote fracture healing. Recent studies focused on the mechanisms of this effect and showed that EPO treatment enhanced the levels of p-JAK2 and p-STAT3, ALP activity, OCN secretion, osteoprotegerin (OPG) and osteopontin (OPN) expression but inhibited RANKL expression in osteoblasts. These effects were abrogated by a JAK2/STAT3 inhibitor (AG490), which indicated that EPO significantly stimulated osteoblast proliferation and differentiation via the JAK2/STAT3 pathway [70]. Naringin, which is the main active ingredient of Chinese herbal medicines, facilitates BMSC proliferation and osteogenic differentiation by inhibiting the JAK2/STAT3 pathway and alleviates bone loss in an ovariectomy (OVX)-induced postmenopausal osteoporosis (PMOP) rat model [71]. Thus far, studies have focused on the role of STAT3 in bone development and bone homeostasis, particularly in response certain stimuli, and a potential treatment strategy or agent showed inconsistent results in terms of the activation of STAT3 and the effect on bone or osteoblast lineage cells, which indicates a nonspecific role of STAT3 in osteogenesis. However, most of these studies demonstrated a parallel association between STAT3 activation and osteogenesis (Table 2; Figs. 2, 3).

### MicroRNAs that regulate osteogenesis via STAT3

MicroRNAs (miRNAs) have been indicated to play an important role in the posttranscriptional regulation of gene expression via translational repression of target mRNAs. An increasing number of miRNAs have been shown to regulate bone homeostasis, and some target STAT3 or its upstream activator JAK2.

JAK2 is an important activator of the STAT3 signalling pathway and has been confirmed as a downstream target gene of miR-135b. With increasing levels of miR-135b, JAK2, p-JAK2 and p-STAT3 levels decreased. MiR-135b inhibition in MC3T3-E1 cells promoted the expression of osteogenesis-related genes and osteoblastic differentiation but reduced apoptosis by upregulating JAK2 expression to activate the JAK2/STAT3 signalling pathway [72]. In apoptotic MC3T3-E1 cells under iron overload conditions, the expression of miR-3074-5p was upregulated. The results of the dual-luciferase reporter assay showed that overexpression of miR-3074-5p caused apoptosis by regulating its downstream target gene Smad4, which blocked the phosphorylation of AKT, ERK and STAT3 [73]. In addition, p-STAT3 also regulates miRNA levels. For example, p-STAT3 directly regulates miR-7-5p expression, which targets cryptochrome circadian regulator 2 (CRY2). Reduced expression of CRY2 stimulated the transcription of P300 by releasing the circadian locomotor output cycles kaput/brain and muscle ARNT-like 1 complex. P300 subsequently promoted the acetylation of histone 3 and the formation of a transcriptional complex with Runx2 to enhance osteogenesis [74] (Table 2; Fig. 2).

However, recent studies have demonstrated that the activation of JAK2/STAT3 signalling is increased by some miRNAs regulation in osteoporosis samples; for example, miR-151a-3p and miR-125b. MiR-151a-3p upregulation significantly decreased the bone mineral density and biomechanical parameters of femurs in OVX rats by targeting SOCS5, and the JAK2/STAT3 pathway is a downstream target of the miR-151a-3p-SOCS5 axis [75]. In vitro studies suggested that miR-125b enhanced the RANKL/OPG ratio and suppressed the levels of BMP2 and Runx2 and cell viability. In a rat OVX model, miR-125b activated the JAK2/STAT3 pathway by altering the downstream target gene TRAF6, thereby reducing bone mineral density and bone biomechanical parameters [76]. Furthermore, miR‐224 may serve as a positive mediator of osteogenic differentiation by deactivating the JAK/STAT3 and Wnt/β-catenin pathways by targeting Rac1 [77] (Table 2; Fig. 3).

In addition to the abovementioned miRNAs that directly or indirectly target STAT3, other studies focusing on cartilage or nonskeletal tissue indicate a more complicated mechanism and network that STAT3 mediates during the development of a series of diseases. It is reasonable to propose that at least some of the miRNAs that affect STAT3 in other tissues are also involved in bone diseases.

### Physiological environment

The duration of hypoxia is crucial for osteogenic differentiation of precursor cells. CoCl_2_-induced hypoxia promoted bone defect healing and upregulated HIF-1α, p-STAT3 and ALP protein expression in the bone defect area, and a STAT3 inhibitor reversed these effects. In vitro, inhibiting STAT3 signalling reduced the hypoxia-induced osteogenic differentiation of BMSCs. Moreover, hypoxia upregulated STAT3 phosphorylation and VEGF expression in MSCs. These results suggest that STAT3 activation is essential for hypoxia-induced osteogenic differentiation in precursor cells and bone defect healing [78] (Table 2; Fig. 2).

## STAT3 signalling pathway and osteoclastogenesis

Bone resorption is dominated by osteoclasts, and these cells attach to the bone surface during the initial stage of bone remodelling in both physiological and pathological conditions. Osteoclastogenesis is a complex process that includes the regulation of various cytokines and the cell–cell fusion of osteoclast precursors. RANKL and macrophage-colony stimulating factor (M-CSF) are indispensable cytokines for osteoclast precursor differentiation and fusion into functional multinucleated giant cells [79]. When RANKL binds to its receptor RANK, osteoclast precursors known as tartrate-resistant acid phosphatase (TRAP)-mononuclear macrophages recruit tumour necrosis factor (TNF) receptor-associated factor 6 (TRAF6). Subsequently, the NF-κB and mitogen-activated protein kinase (MAPK) signalling pathways are activated and further promote the transcription factors nuclear factor of activated T cells (NFATc1) and c-Fos [80]. Thus, the expression of downstream osteoclastic marker genes such as tartrate-resistant acid phosphatase (TRAP), cathepsin K (CTSK), osteoclast-associated receptor (OSCAR), and matrix metalloproteinase-9 (MMP-9) are upregulated and result in osteoclast differentiation and maturation [81, 82]. An increasing number of studies have recently focused on the involvement of other signalling molecules in osteoclastogenesis, particularly in the regulation of osteoclast precursor differentiation and function, including JAK-STAT signalling.

### Dual roles of osteoclast-derived STAT3 in osteoclastogenesis and osteogenesis

Yang et al. investigated the role of STAT3 in osteoclasts in vivo with conditional knockout mice. Analysis of the quantitative microarchitectural parameters of femora from 20-week-old male and female osteoclast-specific STAT3 knockout mice (STAT3^Ctsk^; homozygosis floxed STAT3 and expressing Cre under the Ctsk promoter) showed markedly higher bone mass than their STAT3^fl/fl^ littermates. Far fewer TRAP-positive multinuclear osteoclasts surrounded the trabecular bone of STAT3^Ctsk^ mice than in littermate mice, indicating that the increase in bone mass in STAT3^Ctsk^ mice was mainly due to a direct decrease in bone resorption. The downregulation of osteoclast-specific marker genes, such as OSCAR and dendritic cell-specific transmembrane protein (Dc Stamp), in BMMs from STAT3^Ctsk^ mice suggested that the deletion of STAT3 disrupted osteoclast differentiation, whereas the overexpression of NFATc1 in STAT3-deficient BMMs could rescue the impaired osteoclast differentiation. Mechanistically, STAT3 could directly bind to the promotor of NFATc1 to drive its transcription and osteoclast differentiation [83] (Table 1).

However, another study used mice with conditional knockout of STAT3 in osteoclasts and showed opposite results in terms of bone mass; 8-week-old STAT3-cKO female femurs exhibited significantly lower BMD than those of littermate control females, and there was a lower bone formation rate. Microcomputed tomography (μCT) analysis showed that both male and female STAT3-cKO mice had significantly decreased trabecular bone mass [84]. When STAT3 was knocked down in monocyte/macrophage-like cells (RAW264.7 cells), the expression of early oestrogen-induced gene 1 (EE1G1), NF-kB inhibitor epsilon (NFkBie), and NFATc1 and CTSK signalling were increased, suggesting that STAT3 may regulate osteoclast differentiation and activity through CTSK and its interaction with oestrogen signalling pathways [84].

These results indicate that knocking out STAT3 in osteoclast precursors not only impacts osteoclastic differentiation but also affects osteoblast function, possibly via feedback. In this context, the inconsistent results in bone mass in these two studies, as the authors stated, might be caused by the differences in the genetic backgrounds of the mice and the genotypes of the control mice. However, one major difference that should not be ignored is that the animals in the later study were much younger (8 weeks old) and still in a rapid growth period, during which osteoblast activity plays a more predominant role than that of osteoclasts, while in 20-week-old mice with stable bone remodelling, the function of osteoclasts was more activated than that in the young mice, which may lead to relatively different responses in osteoblasts and osteoclasts to STAT3-cKO; that is, there are more sensitive osteoblasts in younger mice but relatively more sensitive osteoclasts in adult mice to the osteoclast-specific deficiency of STAT3.

### Proteins that regulate osteoclastogenesis via STAT3

#### Cytokines

**Fig. 4:**
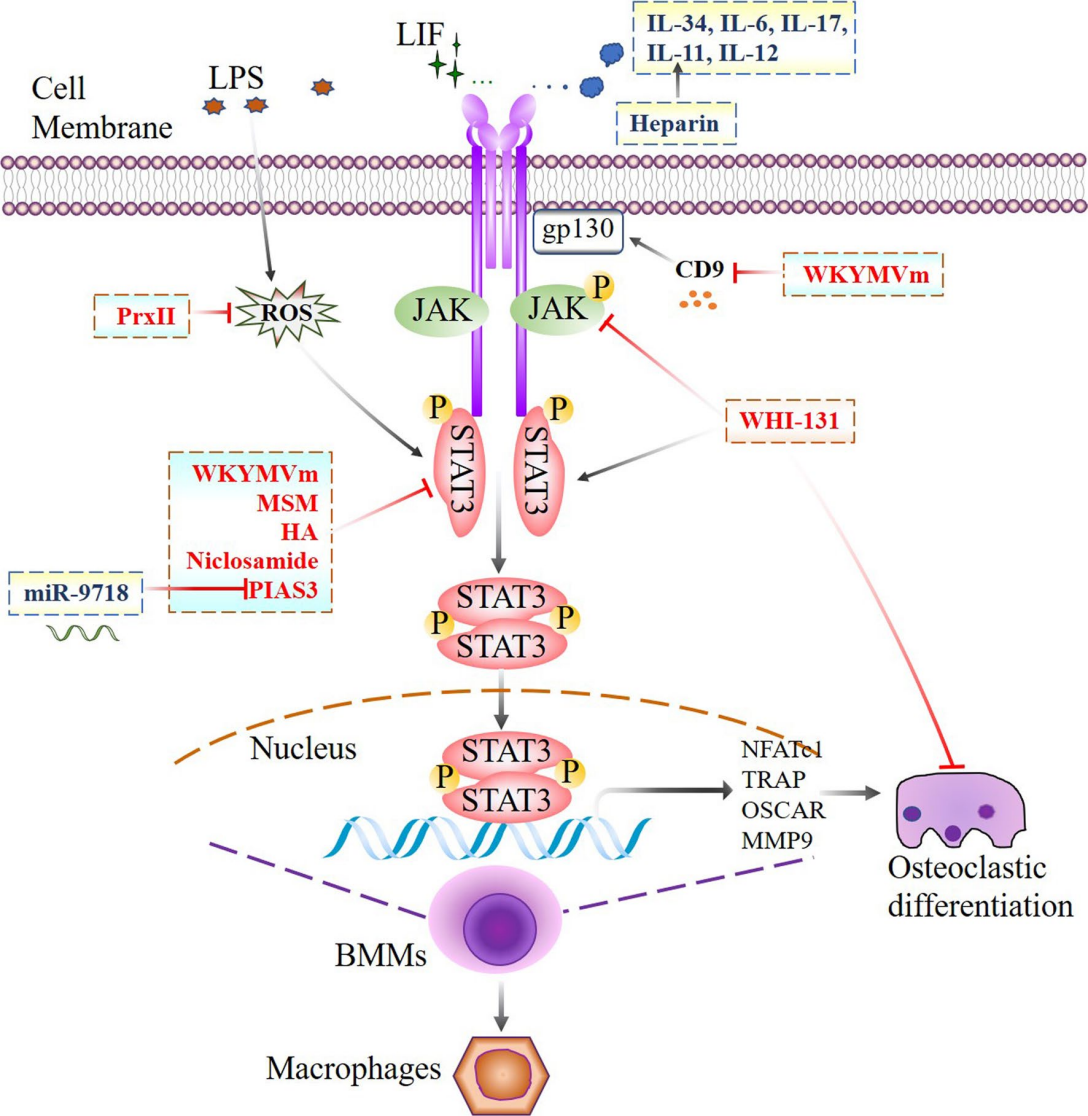
Jak2/STAT3 signal network regulates osteoclastic differentiation. JAK/STAT3 signaling pathway mediated network in regulating osteoclastic differentiation of BMMs with either some members of interleukins or other agents, marked with event-specific colored line or arrows. Those delineated in a rectangle with dotted line indicate functional similarity. Ending with red bars indicate inhibition or downregulation, arrows indicate positive stimulation or upregulation

Previous studies proved that IL-6 induced osteoclastogenesis mainly due to the production of RANKL by osteoblastic cells, which in turn stimulated the differentiation of osteoclast precursors into osteoclasts, thus inducing their maturation and functions. Duplomb et al. demonstrated that IL-6 directly inhibited RANKL-induced osteoclastogenesis by inducing macrophage differentiation. A basal level of p-STAT3 on Ser727 was associated with osteoclastogenesis, and a decrease in Ser727 phosphorylation inhibited osteoclast differentiation. However, Tyr705 phosphorylation prevailed in response to IL-6 stimulation, while Ser727 phosphorylation caused the formation of macrophages instead of osteoclasts [85]. As a novel cytokine involved in bone-related diseases, IL-34 significantly maintained the survival of BMMs and increased the expression of osteoclast-related genes. In addition, higher p-STAT3 levels were observed in BMMs stimulated with IL-34 and RANKL than in those stimulated with RANKL alone, and inhibiting p-STAT3 with AG490 protected mothers against decapentaplegic homologue (Smad7) expression, suggesting that the IL-34/STAT3/Smad7 pathway is critical for BMM proliferation and osteoclastic differentiation [86] (Table 3; Fig. 4).

**Table 3:** Phenotypes of osteoclastic lineage cells with modified STAT3 signaling pathway

Origin	Signaling pathway	Regulating factors	Effect on STAT3 (phosphorylation sites)	Downstream target gene	Phenotypes	Reference
RAW264.7cells and mice BMMs	STAT3/ RANKL	MSM	Decreased STAT3 phosphorylation at Ser727	NFATc1, TRAP, OSCAR, MMP9	Decreased cell viability and TRAP-positive multinucleated cells	[81]
RAW264.7cells and mice BMMs	STAT3, CD9/gp130/STAT3	WKYMVm	Decreased STAT3 phosphorylation at Ser727	NFATc1, c-Fos, MMP9, CTSK	Decreased TRAP-positive multinucleated cells	[82]
ST2 cells and mice BMMs	STAT3	HA	Decreased STAT3 phosphorylation	RANKL	Decreased TRAP-positive multinucleated cells	[97]
RAW264.7cells and mice BMMs	IL-34/STAT3/SMAD7	IL-34	Increased STAT3 phosphorylation	SMAD7	Increased TRAP-positive multinucleated cells	[86]
Mice bone marrow cells	STAT3	Heparin	Increased STAT3-DNA binding and transactivation at Ser727	gp130, RANKL	Increased TRAP-positive multinucleated cells	[93]
RAW264.7cells and BMMs	STAT3	PIAS3	Decreased DNA binding activity of STAT3	c-Fos, NFATc1, OSCAR, RANKL	Decreased TRAP-positive multinucleated cells	[90, 91]
Mice BMMs	STAT3	Niclosamide	Decreased STAT3 phosphorylation at Ser727	c-Fos, NFATc1, TRAP, OSCAR, av/b3 integrin, CTSK	Decreased TRAP-positive multinucleated cells	[95]
Mice BMMs	JAK3/STAT3	WHI-131	Increased STAT3 phosphorylation at Ser727	c-Fos, NFATc1	Decreased TRAP-positive multinucleated cells	[98]
Mice BMMs	STAT3	LIF	Increased STAT3 phosphorylation	CTSK, NFATc1, TRAP, Atp6a3, c-Fos	Increased TRAP-positive multinucleated cells	[108]

#### Cytoplasmic proteins

Peroxiredoxin II (PrxII) is a prime candidate for cell surface receptor-initiated regulation of H2O2 signalling [87]. In vitro, it has been confirmed that lipopolysaccharide (LPS) phosphorylates STAT3 at Tyr705 or Ser727 via reactive oxygen species (ROS), IL-1β, IL-6, and NO production induces osteoclast formation, and this osteoclastogenesis could be inhibited by PrxII. In vivo, under LPS stimulation, PrxII^−/−^ mice showed significantly reduced BV/TV, Tb.Th and Tb.N. PrxII inhibits ROS production and regulates LPS-induced osteoclast formation and bone loss via the STAT3 pathway [88]. PIAS3 binds to STAT3 and blocks DNA binding of STAT3, thereby inhibiting STAT3-mediated gene activation [89]. Transgenic mice overexpressing PIAS3 showed significant increases in BV/TV, Tb.N, Tb.Th and reductions in Tb.Sp. In osteoclast precursors, PIAS3 suppressed RANKL-induced osteoclastogenesis by inhibiting the expression of c-Fos, NFATc1 and OSCAR. In osteoblasts, PIAS3 inhibits the IL-6 cytokine family-gp130-STAT3 signalling, attenuating RANKL expression and inhibiting osteoclastogenesis [90, 91] (Table 3; Fig. 4).

### Peptides that regulate osteoclastogenesis via STAT3

Trp-Lys-Tyr-Met-Val-D-Met-NH2 (WKYMVm) is a synthetic peptide selected from peptide libraries that negatively regulates RANKL- and LPS-induced osteoclastogenesis by directly inhibiting STAT3 phosphorylation at Ser727 and indirectly inhibiting CD9/gp130/STAT3 activity [82]. In vivo, WKYMVm partially rescued LPS-induced bone loss in mice (Table 3; Fig. 4).

### Drugs that regulate osteoclastogenesis via STAT3

Methylsulfonylmethane (MSM) is a naturally occurring sulfur compound with excellent antioxidant and anti-inflammatory properties. STAT3 plays a key role in RANKL-induced osteoclast formation, and MSM attenuates RANKL-induced osteoclastogenesis by blocking the activity of NF-κB and the phosphorylation of STAT3 Ser727 [81]. Long-term heparin exposure is associated with a reduction in bone density [92]. Using an animal model of heparin-induced osteoporosis, Walton et al. found that heparin enhanced both IL-11-induced STAT3-DNA complex formation and transactivation independent of tyrosine or serine phosphorylation of STAT3, leading to the expression of RANKL and gp130, which stimulated osteoclastogenesis and bone loss [93]. Niclosamide (5-chloro-salicyl-(2-chloro-4-nitro) anilide) is a potent inhibitor of STAT3 [94]. In vivo, niclosamide significantly inhibited LPS-induced bone loss in a mouse model. In vitro, niclosamide inhibited RANKL-induced osteoclast differentiation by inhibiting serine-threonine protein kinase phosphorylation, inhibitor of nuclear factor-kappaB (IκB) activation, and STAT3 Ser727 [95]. Hyaluronic acid (HA) is a ubiquitously expressed glycosaminoglycan in the extracellular matrix [96]. HA degradation stimulates the osteoclastogenic potential of osteoclast-supporting cells (ST2 cells) by upregulating RANKL expression and enhancing the expression of vitamin D receptor (VDR) and STAT3 phosphorylation, while stimulating HA attenuated ST2 cell differentiation. These results showed that the accumulation of HA in bone marrow cells may affect RANKL-mediated osteoclast-supporting activity by regulating the VDR and STAT3 signalling pathways [97] (Table 3; Fig. 4).

The small molecule WHI-131, which increases RANKL-induced phosphorylation of STAT3 (Ser727) by inhibiting the expression of JAK3, negatively regulates osteoclast differentiation induced by IL-1, IL-6 or RANKL. Bone histomorphometric analyses showed that WHI-131 treatment alleviated LPS-induced bone loss and notably prevented bone destruction [98] (Table 3; Fig. 4).

Thus, despite a few discrepant data from different groups, most studies confirm a positive association between STAT3 activity and osteoclast function (Fig. 4; Table 3).

### MicroRNAs that regulate osteoclastogenesis via STAT3

Liu et al. identified miR-9718 in primary mouse osteoclasts that promoted osteoclast differentiation by repressing PIAS3 at the posttranscriptional level. In vivo, silencing miR-9718 using a specific antagomir inhibited bone resorption and increased bone mass in OVX mice, indicating that miR-9718 played an important role in osteoclast differentiation by targeting PIAS3 both in vitro and in vivo [99] (Table 3; Fig. 4).

### Periprosthetic osteolysis

The inflammatory response to wear debris is an important factor in the development of periprosthetic osteolysis. It was confirmed that polymethylmethacrylate and titanium significantly inhibited IL-6-induced STAT3 activation in osteoclast precursors, suggesting that the mechanism of wear debris in periprosthetic osteolysis may be through the inhibition of anti-osteoclastogenic cytokine signalling [100] (Table 3; Fig. 4).

## Cell–cell interactions via STAT3 activation

### Haematopoietic cells and osteoclasts

**Fig. 5:**
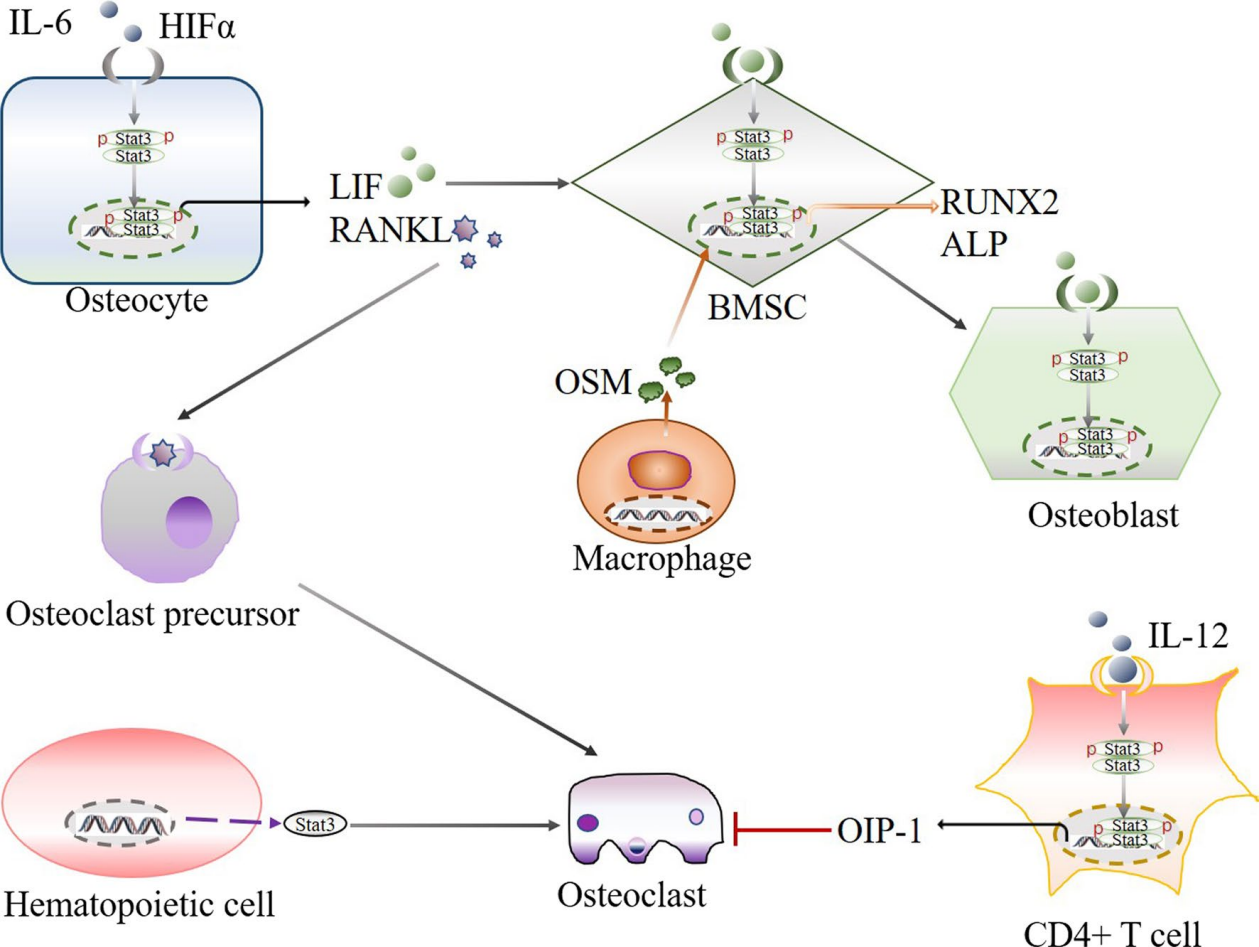
STAT3 signal mediated cellular interaction within osteocyte, BMSC, osteoblast, osteoclast, osteoclast, precursor, macrophage, hematopoietic cell and CD4 + T cell through regulating osteogenesis- and osteoclastogenesis-related factors. Arrows indicate positive stimulation or upregulation

Interestingly, haematopoietic cell-specific knockout of STAT3 in transgenic mice not only induced hyperproliferation of the myeloid lineage but also led to reductions in BV and Tb.Th and Tb.N by 69.5%, 84.6% and 77.0%, respectively. In addition, increased numbers of Mac1 + cells and c-kit + cells were also identified, suggesting an increased number of osteoclast precursors in STAT3-deficient mice. These results showed that haematopoietic cell-derived STAT3 plays a negative role in regulating osteoclastogenesis [101] (Fig. 5).

### Immune cells & osteoclasts

Osteoclast formation is regulated by a variety of cell types and their products, such as immune cells that produce IL-12, which has been confirmed to be a potent inhibitor of osteoclast formation. Human osteoclast inhibitory peptide-1 (OIP-1/hSca) is a Ly-6 gene family member, and IL-12 stimulates OIP-1 expression through STAT-3 activation in CD4 + T cells. Chromatin immunoprecipitation (ChIP) assays confirmed STAT-3 binding to the OIP-1 promoter element in response to IL-12 stimulation [102].

In addition, IL-17 is produced by Th17 cells, which may be involved in the pathogenesis of bone loss. Oestrogen (E2) increased the differentiation of Th17 cells and upregulated STAT3 expression. IL-17 neutralizing antibody treatment of OVX mice significantly increased BV/TV, Tb.N. and connection density (Conn. Dn), and reduced Tb.Sp, trabecular pattern factor (Tb.Pf), the structure model index (SMI) and TRAP-positive cells [103] (Fig. 5).

### Osteoblast lineage and osteoclasts

Osteoblasts can differentiate into osteocytes, which secrete M-CSF and RANKL in the bone matrix, regulating osteoclast formation.

Hypoxia‐inducible factor‐1α (HIF‐1α) is one of the major regulatory factors of the hypoxia response and plays an important role in inflammation, cell proliferation and bone metabolism [104]. Overexpression of HIF‐1α in osteocyte‐like MLO‐Y4 cells upregulated RANKL levels through the phosphoproteins JAK2 and STAT3 and facilitated the differentiation of RAW264.7 osteoclast precursors into osteoclasts [105].

IL-6 enhances osteocyte-mediated osteoclastic differentiation and osteoclastogenesis by activating the JAK2-STAT3 pathway, which is required for RANKL induction by IL-6/IL-6R [106]. IL-6-type cytokines also enhance osteoclast formation by activating the gp130 receptor subunit on stromal/osteoblastic cells, which leads to STAT3-mediated expression of RANKL [107].

LIF secretion by osteocytes can increase the tartrate-resistant acid phosphatase activity of BMM-derived osteoclasts and enhance alkaline phosphatase activity during the osteogenic differentiation of BMSCs. Mechanistically, LIF increases the phosphorylation of STAT3, which is associated with osteoclast and osteoblast formation [108] (Fig. 5).

### Monocytes/macrophages and MSCs

It has been reported that monocytes induce STAT3 activation in human MSCs to promote osteoblast formation. Using in vitro cell cultures of human bone marrow-derived MSCs, it was shown that monocytes/macrophages effectively induced MSC differentiation into osteoblasts through cell contact, and the former produced oncostatin M to activate STAT3 in the latter. The phosphorylation of STAT3 in MSCs upregulated RUNX2 and ALP expression [108] (Fig. 5).

In contrast to studies that focused on single-cell functions regarding the role of STAT3 in osteogenesis and osteoclastogenesis, these data showed cellular interactions and provided valuable insight into STAT3-mediated events in bone, which should be further examined in future studies.

## Conclusion

Although the data we reviewed are somewhat inconsistent in terms of the role of STAT3 in events involved in bone remodelling and osteoporosis, most evidence supports a positive effect of STAT3 on both osteogenesis and osteoclastogenesis in vivo, as cell-specific deficiency in either osteoblastic or osteoclastic linage cells impaired differentiation or even cell function. However, these complicated cellular interactions, either during the development of bone and related diseases, including osteoporosis, or under certain stimuli, make it difficult to draw firm conclusions with respect to the role of STAT3 in these processes; otherwise, we could state that the role of STAT3 play in these eventsis cell- and stimulation-specific. In this context, more studies should be performed to determine the cellular network in which STAT3 participates, thereby uncovering whether and how cell-specific alterations in STAT3 activity could affect the development of osteoporosis or whether systematically silencing or overactivating STAT3 could be a potential strategy for osteoporosis treatment. On the other hand, further investigation of the role of STAT3 in the development of subtypes other than oestrogen deficiency-induced osteoporosis is also important. Finally, STAT3-mediated cell–cell interactions involve a wide range of nonbone cells, which enlarges the window to show how these factors interact with bone cells during osteoporosis and should be examined in future studies.

## Data Availability

Not applicable.

## Abbreviations

STAT3: Signal transducer and activator of transcription 3
BMSCs: Bone marrow stromal cells
BMM: Bone-marrow macrophages
RANKL: Receptor activator of nuclear factor-kb ligand
FDA: Food and Drug Administration
SOCS: Suppressors of cytokine signaling
PTPs: Protein tyrosine phosphatases
SH2: Src homology 2
Tyr705: Tyrosine 705
Ser727: Serine 727
IL: Interleukin
gp130: Glycoprotein 130
SHP-2: Src homology phosphatase-2
BV: Bone volume
Tb.N: Trabecular number
Tb.Th: Trabecular thickness
Tb.Sp: Trabecular separation
Ct.Th: Cortical thickness
Conn.Dn: Connection density
Tb.Pf: Trabecular pattern factor
SMI: Structure model index
PIAS3: Protein inhibitor of activated STAT3
OVX: Ovariectomy
PC1: Polycystin-1
EPO: Erythropoietin
NDRG2: N-myc downstream regulator gene 2
BMP2: Bone morphogenetic protein 2
Ror2: Orphan receptor 2
Runx2: Runt-related transcription factor 2
OPG: Osteoprotegerin
OSX: Osterix
ALP: Alkaline phosphatase
OCN: Osteocalcin
PKD: Protein kinase D
CUEDC2: CUE domain-containing 2
LepR: Leptin receptor
LIF: Leukemia inhibitory factor
RPN2: Ribophorin II
mTOR1: Mechanistic target of rapamycin complex 1
miRNAs: MicroRNAs
CRY2: Cryptochrome circadian regulator 2
M-CSF: Macrophage-colony stimulating factor
TRAP: Tartrate-resistant acid phosphatase
TNF: Tumor necrosis factor
TRAF6: TNF receptor-associated factor 6
NFATc1: Nuclear factor of activated T cells
μCT: Micro-computed tomography
MAPK: Mitogen-activated protein kinases
OSCAR: Osteoclast-associated receptor
PrxII: Peroxiredoxin II
LPS: Lipopolysaccharide
ROS: Reactive oxygen species
WKYMVm: Trp-Lys-Tyr-Met-Val-D-Met-NH2
MSM: Methylsulfonylmethane
HA: Hyaluronic acid
IL-34: Interleukin-34
PIAS3: Protein inhibitor of activated STAT3
Niclosamide: 5-Chloro-salicyl-(2-chloro-4-nitro) anilide
EE1G1: Estrogen-induced gene 1
NFkBie: NF-kB inhibitor epsilon
RAS: Renin-angiotensin system
ERK: Extracellular signal-regulated kinase
PTPRD, PTPRT, PTPRK: PTP receptor-type D, T and K
PTPN9: PTP: non-receptor type 9
PTPN2: PTP non-receptor type 2
